# Functional genomics analysis reveals the biosynthesis pathways of important cellular components (alginate and fucoidan) of *Saccharina*

**DOI:** 10.1007/s00294-017-0733-4

**Published:** 2017-08-19

**Authors:** Shan Chi, Tao Liu, Xumin Wang, Ren Wang, Shanshan Wang, Guoliang Wang, Guangle Shan, Cui Liu

**Affiliations:** 1 Ocean University of China, Qingdao, Shandong Province People’s Republic of China; 2 Qingdao Haida BlueTek Biotechnology Co., Ltd, Qingdao, Shandong Province People’s Republic of China; 3 CAS Key Laboratory of Genome Sciences and Information, Beijing Key Laboratory of Genome and Precision Medicine Technologies, Beijing Institute of Genomics, Chinese Academy of Sciences, Beijing, People’s Republic of China; 4 Beijing Key Laboratory of Functional Genomics for Dao-di Herbs, Beijing Institute of Genomics, Chinese Academy of Sciences, Beijing, People’s Republic of China; 5 University of Chinese Academy of Sciences, Beijing, People’s Republic of China

**Keywords:** Alginate, Fucoidan, Brown algae, *Saccharina*, Biosynthesis pathway

## Abstract

Although alginate and fucoidan are unique cellular components and have important biological significance in brown algae, and many possible involved genes are present in brown algal genomes, their functions and regulatory mechanisms have not been fully revealed. Both polysaccharides may play important roles in the evolution of multicellular brown algae, but specific and in-depth studies are still limited. In this study, a functional genomics analysis of alginate and fucoidan biosynthesis routes was conducted in *Saccharina*, and the key events in these pathways in brown algae were identified. First, genes from different sources, including eukaryotic hosts via endosymbiotic gene transfer and bacteria via horizontal gene transfer, were combined to build a complete pathway framework. Then, a critical event occurred to drive these pathways to have real function: one of the mannose-6-phosphate isomerase homologs that arose by gene duplication subsequently adopted the function of the mannose-1-phosphate guanylyltransferase (MGP) gene, which was absent in algal genomes. Further, downstream pathway genes proceeded with gene expansions and complex transcriptional mechanisms, which may be conducive to the synthesis of alginate and fucoidan with diverse structures and contents depending on the developmental stage, tissue structure, and environmental conditions. This study revealed the alginate and fucoidan synthesis pathways and all included genes from separate phylogenetic sources in brown algae. Enzyme assays confirmed the function of key genes and led to the determination of a substitute for the missing MPG. All gene families had constitutively expressed member(s) to maintain the basic synthesis; and the gene function differentiation, enzyme characterization and gene expression regulation differences separated brown algae from other algae lineages and were considered to be the major driving forces for sophisticated system evolution of brown algae.

**Electronic supplementary material:**

The online version of this article (doi:10.1007/s00294-017-0733-4) contains supplementary material, which is available to authorized users.

## Introduction

Alginate and fucoidan are the major components of brown algal cell walls, functioning as support molecules and fillers, respectively (Kloareg and Quatrano 1988; Morya et al. 2012; Bartsch et al. 2008). These polysaccharides are thought to have important biological significance in the evolution of special features of multicellular organisms, including mechanical shear resistance, external tissue damage reduction, and enhanced flexibility (Bartsch et al. 2008). They also have various biological and physical features, such as emulsifying, anticoagulant, antitumor, and antiviral effects, enhanced immunity, and roles in maintaining blood pressure (Indergaard and Ostgaard 1991; Teas 1983; Smit 2004; Mayer and Hamann 2005), traits that promote their wide use in food products, drugs, cosmetics, fertilizers, and scientific research (Cunha and Grenha 2016; Tseng 1987; McHugh 2003; De Roeck-Holtzhauer 1991; Thomas 2000). Despite the physiological and economic importance of alginate and fucoidan, information on their biosynthesis pathways in brown algae is scarce: the complete pathways remain unknown, only a few genes have confirmed functions, and regulatory mechanisms have been poorly studied. Therefore, further studies on their pathway evolution, gene function and expression regulatory mechanisms are warranted.

Brown algae are among the most important groups of macroalgae in marine ecosystems of cold, temperate, and tropical coastal zones owing to their high species richness, biomass, and economic value (Liu and Pang 2015; Charrier et al. 2012). Brown algae diverged from other major heterokont organisms (or stramenopiles) over one billion years ago (Yoon et al. 2004; Baldauf 2008). Among the secondary endosymbiotic algae, brown algae exhibit many unique and interesting developmental, physiological, and metabolic features compared with other taxa (e.g. diatoms), and are the only algae with sophisticated multicellularity (Cock et al. 2014; Niklas and Newman 2013; Knoll 2011).

*Saccharina*, one of the most important genera of brown algae, is currently widely farmed in China and other East Asian countries. Its production has rapidly increased from 5 thousand tons in 1950 (wild resources) to 7.65 million tons in 2014 (mainly aquaculture products), and has become one of the major global species of aquatic plants (Tseng 2001; FAO 2016). The growing demand for *Saccharina* can be attributed to its special carbohydrate metabolism and products, such as alginate, fucoidan, iodine, mannitol, and other economically valuable elements (Zia et al. 2015; Klettner 2016; Fitton et al. 2015; Zhang et al. 2011; Dar et al. 2002). The life history of *Saccharina* contains a series of succession processes from a single cell (meiospore) to a multicellular filamentous (gametophyte, n) and eventually a large parenchyma individual (sporophyte, 2n) (Bartsch et al. 2008). *Saccharina*, with a unique heteromorphic alternation of generations, is quite different from its close relatives in the genus *Ectocarpus*, which lack the parenchyma stage (Cock et al. 2014). At the same time, tissue differentiation is observed in the mature sporophyte of *Saccharina* individuals. Previous biochemical studies demonstrated that the contents and chemical structure of alginate and fucoidan vary among different developmental stages (e.g. spring juvenile algae and summer mature algae) and tissue structures (e.g. blade base, tip, fascia, and pleat) (Skriptsova et al. 2010; Morya et al. 2012; Obluchinskaia et al. 2002). Therefore, an important question is whether the complex regulatory mechanisms of alginate and fucoidan synthesis impact the structural evolution between filamentous brown algae (*Ectocarpus*) and heteromorphic haploid-diploid algae (*Saccharina*).

Recent structural and functional genomics studies using *Ectocarpus siliculosus* and *Saccharina japonica* analyzed the synthesis pathways of these components and provided clues regarding the adaptation of brown algae to the highly variable environment (Cock et al. 2010; Ye et al. 2015). Parts of the brown algal alginate and fucoidan biosynthesis routes and genes had been predicted based on the synthetic pathways of bacterial alginate and animal fucoidan, respectively (Michel et al. 2010). Among these predicted genes in brown algae, only one GDP-mannose 6-dehydrogenase (*GMD1*) from *E. siliculosus* (Tenhaken et al. 2011) and several mannuronate C5-epimerases (*MC5Es*) from *Laminaria digitata* (Nyvall et al. 2003) had been isolated and confirmed. The other predicted genes have not been functionally verified. In addition, one shared gene mannose-1-phosphate guanylyltransferase (*MPG*) of these two pathways, that encodes the key enzyme that catalyzes the conversion of mannose-1-phosphate to GDP-mannose (Akutsu et al. 2015), has not been annotated in the brown algae genome (Gurvan et al. 2010; Ye et al. 2015). Furthermore, there are also no *MPG* homologs in diatoms *Thalassiosira pseudonana* and *Phaeodactylum tricornutum* (Armbrust et al. 2004; Bowler et al. 2008). Therefore, it is not clear which gene functions similarly to *MPG* in brown algae and whether the biosynthesis pathways in brown algae and bacteria differ.

Comprehensive bioinformatics and phylogenetic analyses of numerous brown algae, including one of the most popular Chinese *Saccharina* varieties “Rongfu” (Zhang et al. 2011), integrated with enzyme function assay, have revealed the biosynthesis routes for brown algal alginate and fucoidan. Droplet digital PCR and transcriptome sequencing of different *Saccharina* tissues from all life-history stages (including the sporophyte and gametophyte generations) were conducted to understand gene expression regulatory mechanisms involved in these pathways. The results of this study will expand our understanding of the regulatory mechanism of carbohydrates in brown algae and provide a basis for improved algae utilization and breeding.

## Methods

### Algal sample collection

Preserved *S. japonica* haploid gametophytes (gametocyte, male and female gametophytes) were available as laboratory cultures and obtained from our Laboratory of Genetics and Breeding of Marine Organisms. Fresh samples of the high-yielding *Saccharina* cultivar “Rongfu” (Zhang et al. 2011) sporophytes (juvenile stage; uneven stage; blade base, and fascia of smooth stage; blade tip, pleat, base, and fascia of adult stage; and blade pleat of mature stage) were collected from east China (Rongcheng, Shandong Province, 37°8′53″N, 122°34′33″E). These samples were used for genome re-sequencing, transcriptome sequencing and droplet digital PCR analysis.

### Genome re-sequencing

Three paired-end libraries and three mate-paired libraries were constructed according to Illumina standard operating procedure. Sequencing of each library was performed on an Illumina HiSeq 2000 instrument to produce the raw data. Then, low-quality and short reads were filtered out to obtain a set of usable reads. Clean seaweed materials were used for DNA extraction according to the cetyltrimethyl ammonium bromide (CTAB) method as described previously (Sun et al. 2011; Guillemaut and Drouard 1992). The reads were then assembled into contigs using SOAPdenovo (Li et al. 2009) with varying parameters, and mate-paired relationships between the reads were used to construct scaffolds.

### Transcriptome sequencing

Total RNA was extracted using an improved CTAB method (Gareth et al. 2006). cDNA library construction and sequencing were performed by the BGI (Shenzhen, China) on Illumina (San Diego, USA) HiSeq instruments in accordance with the manufacturer’s instructions. Fragment size selection was performed using agarose gel electrophoresis, from which fragments of 200–250 bp were extracted. Strict reads filtering was performed before the assembly. Pair-end reads with primer or adapter sequences were removed. Reads with more than 10% of bases below Q20 quality or more than 5% of bases as unknown nucleotides (Ns) were filtered from total reads. De novo assembly was carried out using SOAPdenovo-Trans (Li et al. 2009) (http://soap.genomics.org.cn/SOAPdenovo-Trans.html). Gapcloser was then used for gap filling of the scaffolds.

### Sequence analysis and phylogenetic tree construction

In the present study, genes were identified by analyzing transcriptomic and genomic sequencing data of *S. japonica* (Tao Liu, unpublished data), as well as the species whose genome and transcriptome data were sequenced and published in OneKP (www.onekp.com) or NCBI. Matching sequences were manually checked for accuracy with the corresponding known cDNA sequences. All downloaded sequences are listed in Table S1. The sequences were aligned using ClustalX 1.83 software (Thompson et al. 1997). MrBayes 3.1.2 software was used to construct the amino acid phylogenetic trees (Ronquist and Huelsenbeck 2003). The posteriori probability is based on the Metropolis–Hastings–Green algorithm through four chains (Markov Chain Monte Carlo, MCMC) with the temperature set to 0.2 °C. The chains would be run for 10,000,000 cycles (Ronquist and Huelsenbeck 2003; Posada and Crandall 1998). In the MCMC analysis, random trees were constructed, and one tree every 1000 generations was saved. After discarding the aging 25% of samples of all these trees, the residual samples were used to construct a consensus tree and then Tree View v.1.6.5 software was used to render the tree (Page 1996).

### Protein purification and enzyme kinetic assays

Genes were synthesized (Shanghai Xuguan Biotechnological Development Co,. LTD) and cloned in pET32a to construct recombinant plasmids. The plasmids were transformed into *E. coli* BL21 (DE3) to overexpress recombinant proteins. IPTG (0.1 mM) was added to induce over-expression of the target proteins, and bacteria were incubated for 12 h at 16 °C. His-Binding-Resin was used according to the manufacturer’s instructions (www.yuekebio.com) to separate the target proteins. According to the methods described in previous studies (Maruta et al. 2008; Sousa et al. 2007; Richau et al. 2000; Tenhaken et al. 2011), enzyme reaction conditions were optimized by temperatures, pH values, and metal ions. Four replicates were analyzed for each condition to ensure the reproducibility of the experimental results. All the data were subjected to one-way analysis of variance (one-way ANOVA) followed by a Student’s *t* test.

### Droplet digital PCR and analysis

To detect the influences of abiotic factors, the gametophyte samples of *S. japonica* were cultured under different temperatures (control: 8 °C; hyperthermia: 18 °C), salinity (control: 30‰; hyposaline: 12‰), and irradiance (control: 30 µmol photons/m^2^ s for 12 h; Darkness: 72 consecutive hours of darkness). Various *Saccharina* gametophyte samples (gametocyte, male and female gametophytes) and tissue structures of sporophytes (juvenile stage; uneven stage; blade base, and fascia of smooth stage; blade tip, pleat, base, and fascia of adult stage; and blade pleat of mature stage) were used to analyze relative gene expression. Each 25 μL reaction setup contained 1 × Droplet PCR Supermix (Bio-Rad), 900 nmol of each primer, 250 nmol of the probe, and 3 μL of sample DNA. The primers and probes used are listed in Table S4. The reaction mixture was mixed with droplet generation oil (20 μL mixture + 70 μL oil) via microfluidics in the Droplet Generator (Bio-Rad). Following droplet generation, the water-in-oil droplets were transferred using a multichannel pipette to a standard 96-well PCR plate, which was heat sealed with a foil plate seal (Bio-Rad) and placed on a Bio-Rad CFX96 thermocycler (ramping speed at 2.5 °C s^−1^) for PCR amplification using the following conditions: 10 min at 95 °C, followed by 40 cycles of 30 s at 94 °C and 60 s at 60 °C, followed by a 10 min hold at 98 °C. Upon PCR completion, the plate was transferred to a Droplet Reader (Bio-Rad) for automatic measurement of fluorescence in each droplet in each well (approximately 2 min per well), with the RED (rare event detection) setting. The results represent mean values of three ddPCR analysis.

## Results

### The rise of the alginate and fucoidan pathways: complex endosymbiotic gene transfer (EGT) and horizontal gene transfer (HGT) origins

Novel transcriptomic sequencing data obtained for 21 rhodophytes and 19 Phaeophyceae marine species (OneKP database), re-sequencing genomic data for *Saccharina* (“Rongfu”), and publicly available genomic data for algae were used to identify genes involved in alginate and fucoidan biosynthesis, and 200 new full-length candidate genes from algae were detected (Table S1). There were 6 and 8 genes (or families) involved in alginate and fucoidan biosynthesis in algae, respectively. Three shared upstream genes, mannose-6-phosphate isomerase (*MPI*), phosphomannomutase (*PMM*), and *MPG*, are involved in converting fructose-6-phosphate into GDP-mannose. Then, GDP-mannose flows in two separate directions: the alginate biosynthesis route involves GDP-mannose/UDP-glucose 6-dehydrogenase (*GMD/UGD*), mannuronan synthase (*MS*), and *MC5E*; the fucoidan biosynthesis route involves GDP-mannose 4,6-dehydratase (*GM46D*), GDP-fucose synthetase (*GFS*), fucosyltransferase (*FS*), and sulfotransferase (*ST*) in a de novo pathway and fucokinase (*FK*), and GDP-fucose pyrophosphorylase (*GFPP*) in an alternative salvage pathway.

**Fig. 1:**
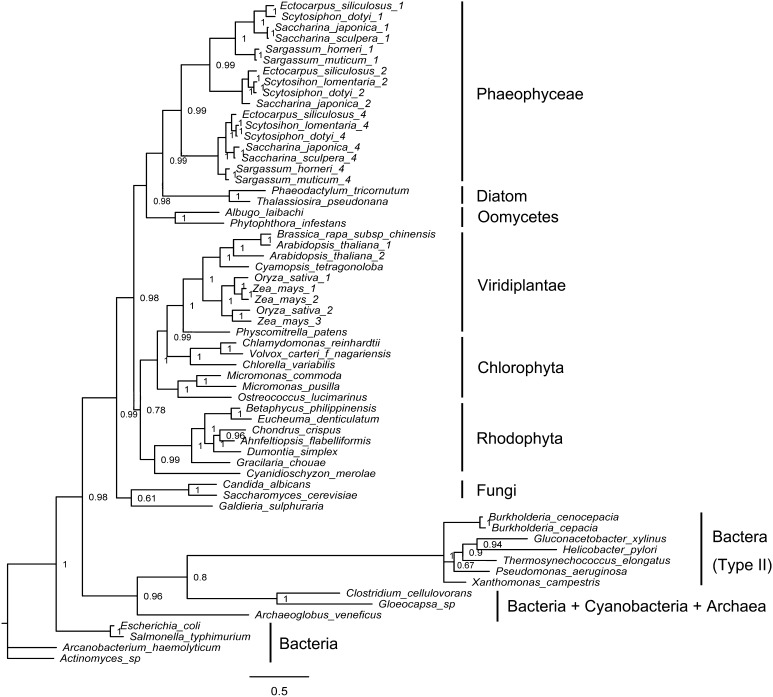
Bayesian phylogenetic tree based on the translated amino acids of mannose-6-phosphate isomerase (MPI) with bootstrap values (when >50%) indicated at the *nodes*. All eukaryotic algal *MPIs* (including those of brown algae, diatoms, red algae, and green algae) originated from eukaryotic hosts. Brown algae *MPIs* underwent gene duplication in their common ancestor. Bacterial type II *MPIs* encode bifunctional enzymes with MPI and MPG activities. All MPI sequences were obtained from GenBank or OneKP databases (Table S1)

**Fig. 2:**
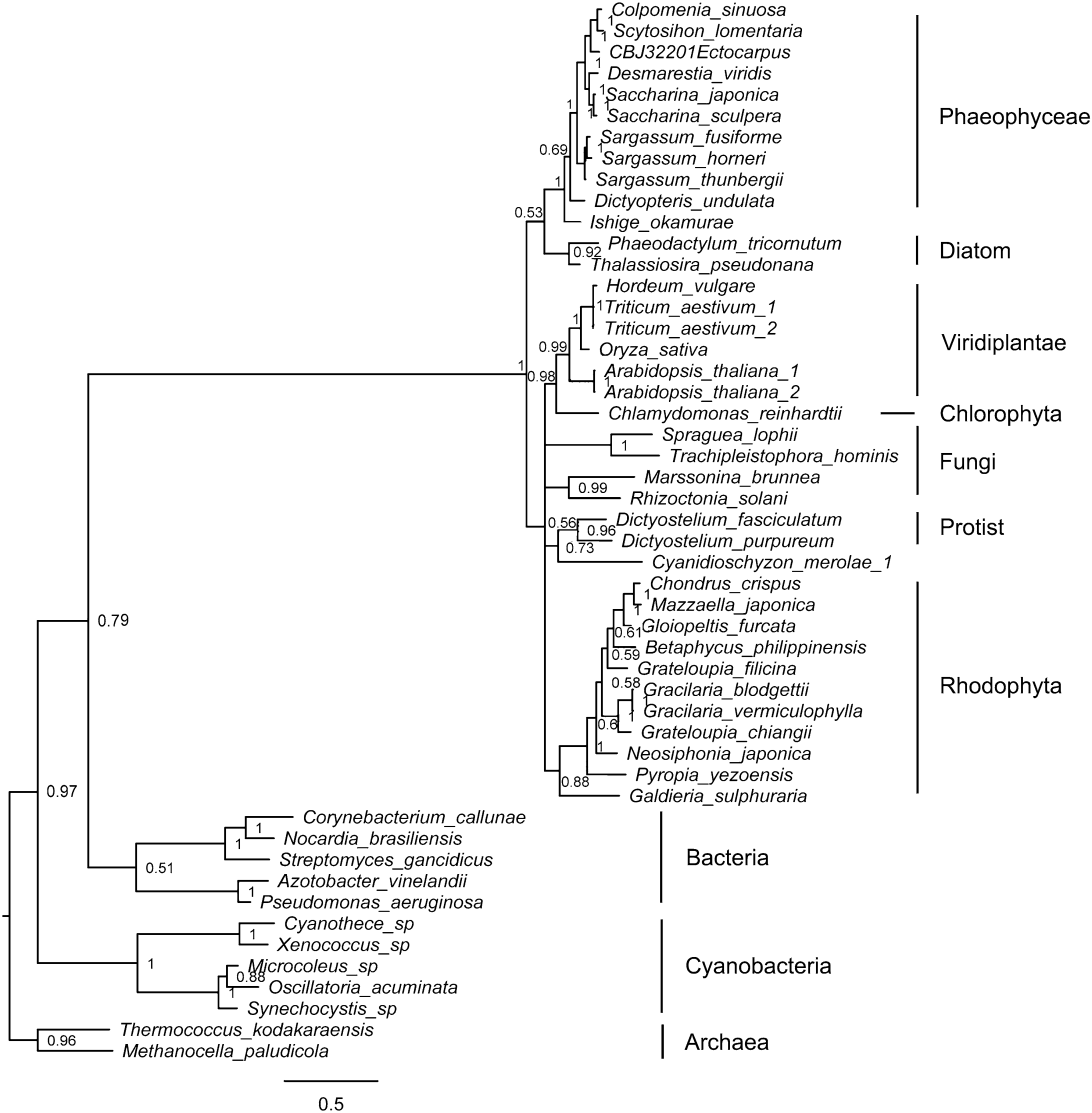
Bayesian phylogenetic tree based on the translated amino acids of phosphomannomutase (PMM) with bootstrap values (when >50%) indicated at the *nodes*. All eukaryotic algal *PMMs* (including those of brown algae, diatoms, red algae, and green algae) originated from eukaryotic hosts. All PMM sequences were obtained from GenBank or OneKP databases (Table S1)

**Fig. 3:**
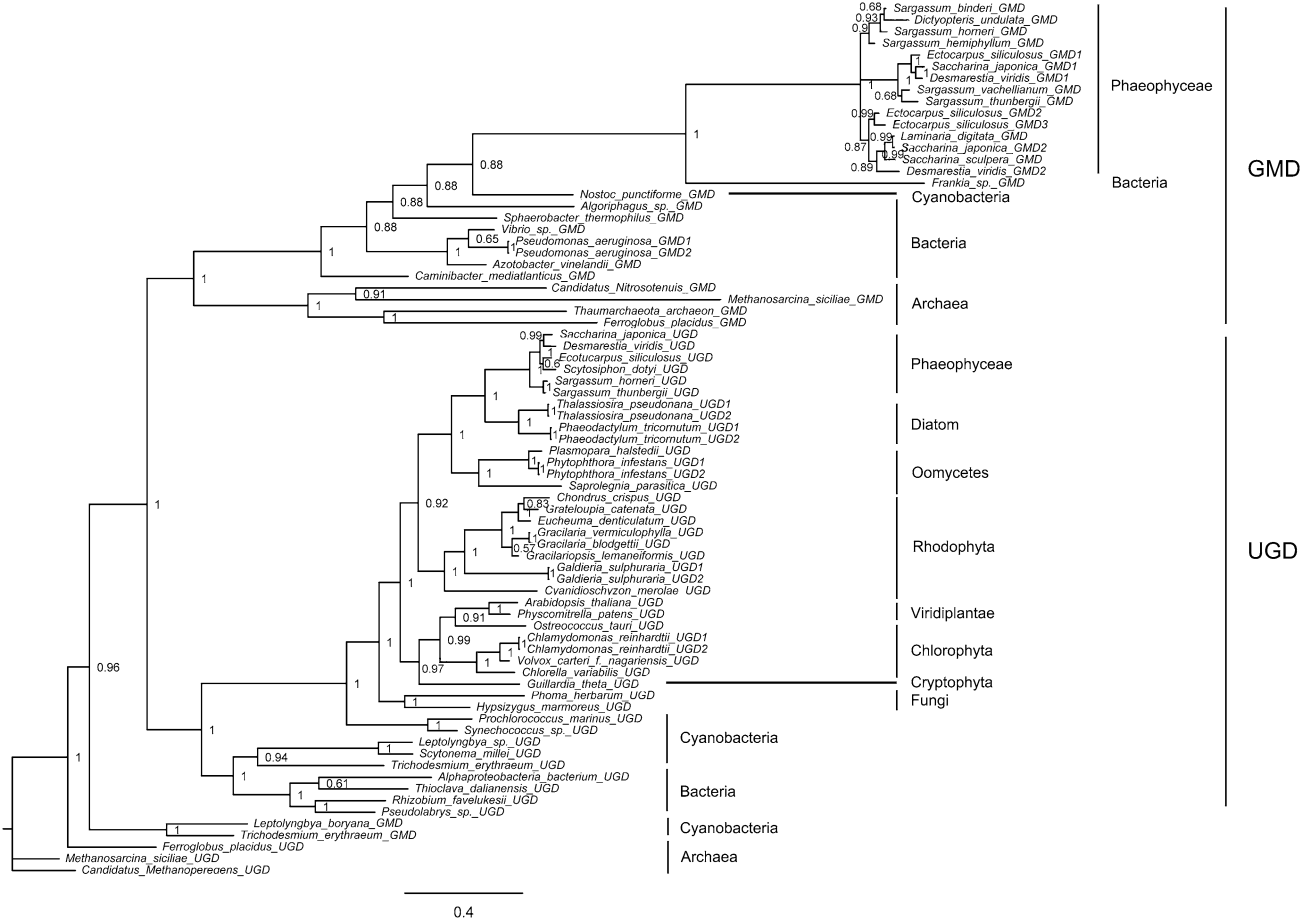
Bayesian phylogenetic tree based on the translated amino acids of GDP-mannose/UDP-glucose 6-dehydrogenases (GMD/UGD) with bootstrap values (when >50%) indicated at the nodes. Brown algal *GMD* was inherited from bacteria by HGT. *UGD* may originate from eukaryotic hosts: red algae acquired *UGD* from primary endosymbiotic hosts, while diatoms and brown algae acquired from secondary endosymbiotic hosts. All GMD/UGD sequences were obtained from the GenBank or OneKP databases (Table S1)

**Fig. 4:**
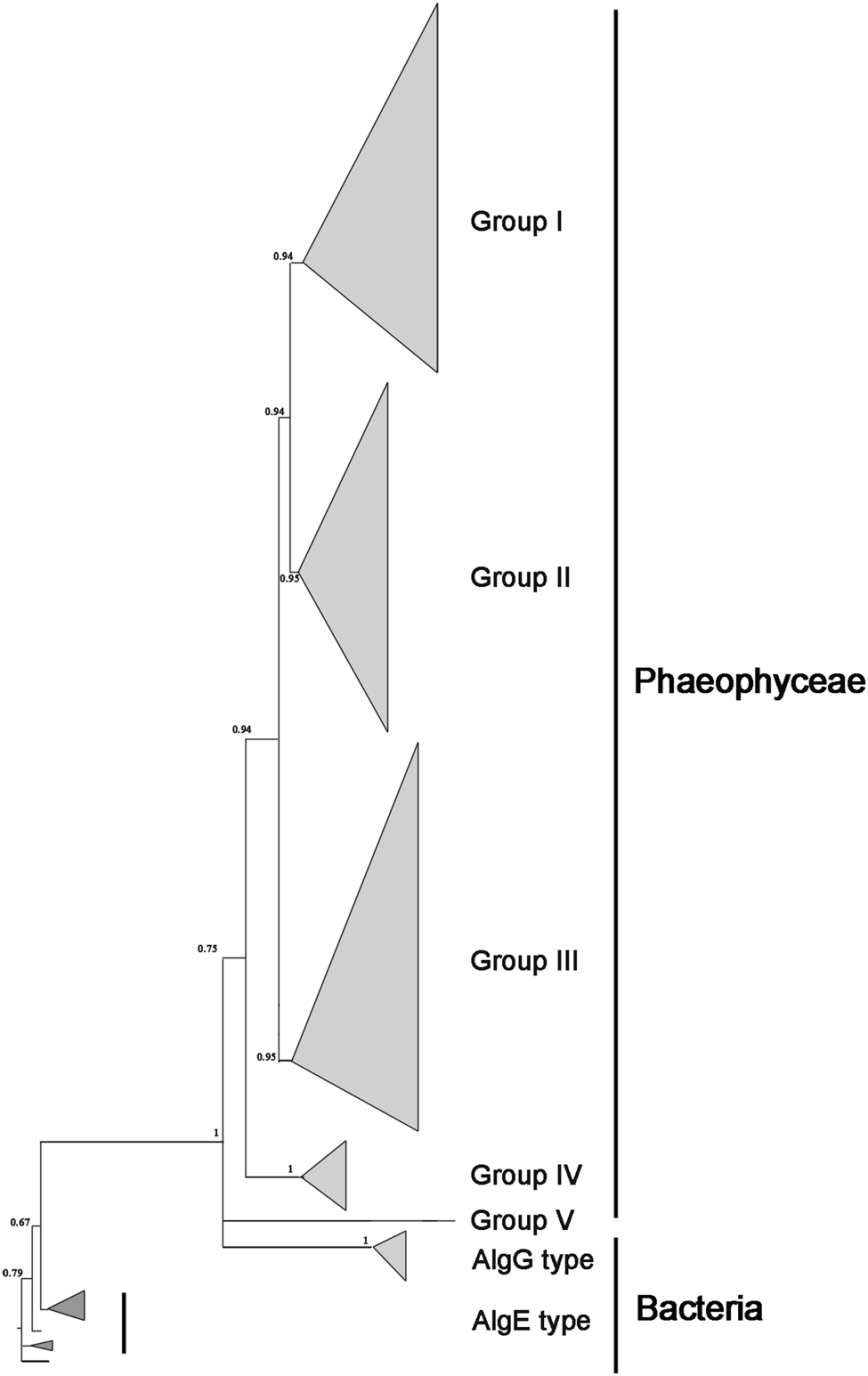
Summary of a Bayesian tree based on mannuronate C5-epimerases (MC5E). Brown algae acquired *MC5E* from bacteria via HGT and the gene subsequently duplicated to form five groups (I–V). The detailed phylogenetic tree is shown in Figure S4

Phylogenetic trees based on full-length amino acid sequences of alginate and fucoidan biosynthesis-related genes from archaeal taxa, bacteria, cyanobacteria, fungi, oomycetes, protozoa, tracheophytes, and eukaryotic algae were constructed using Bayesian methods (only representative candidates were included). Based on these trees, the alginate and fucoidan biosynthesis genes of brown algae have two different origins (Figs. 1, 2, 3, 4, S1 and S2). *MPI, PMM, GM46D,* and *GFS* were distributed among diverse algae, and were inherited from eukaryotic hosts during secondary endosymbiosis (Figs. 1, 2; Figs. S1, and S2). With respect to the *GMD*/*UGD* (Fig. 3) gene family, *GMD* was only found in brown algae with a bacterial HGT origin, and *UGD* was distributed widely and inherited from eukaryotic hosts during endosymbiosis. *MC5E,* like *GMD,* was only found in brown algae and originated from bacteria via HGT (Fig. 4).

Brown algae genomes contained three full-length *MPI* genes (*MPI1*, *MPI2,* and *MPI4* according to the naming mode for *E. siliculosus*) and two fragments (one N-terminal and one C-terminal, jointly named *MPI3*) (Table S1). Within the consensus tree, all MPIs from Phaeophyceae and diatom species (both secondary endosymbiosis lineages) formed a group within a large clade including oomycete MPIs (Fig. 1). The shared lineage including Phaeophyceae and oomycete genes suggests that they are derived from eukaryotic hosts. In the Phaeophyceae branch, each homolog of MPIs (from different species) formed a distinct cluster, suggesting that gene duplication occurred in the common ancestor of Phaeophyceae. *MPI4* formed the sister group to the group including *MPI1* and *MPI2*, suggesting that *MPI4* diverged earlier than these two genes. There were multi-copy *MPIs* in Phaeophyceae species, different from the single copy observed in other algal groups (including other algal phyla and other stramenopiles, such as diatoms), indicating that *MPI*s may have a unique function in Phaeophyceae in alginate and fucoidan synthesis.

There is one *PMM* (GenBank No. CBJ32201) and one *PMM/*phosphoglucomutase (*PGM*) gene (GenBank accession number FN648060.1) in the *E. siliculosus* genome. However, only the full-length homolog of *PMM* was detected in our *Saccharina* genome data and transcriptome data for other brown algae. In addition, the NCBI published *PMM/PGM* gene in *S. japonica* (GenBank accession number KP772272.1) does not contain the phosphomannomutase domain predicted by “Conserved Domain Search” (NCBI service) (Figure S3). Therefore, these brown algal *PMM/PGM* sequences were not considered in this study. A phylogenetic analysis showed that all brown algal PMMs formed a group within a large lineage including PMMs of other eukaryotic algae, fungi, and protists, and clustered into a single group with PMMs of bacteria (not cyanobacteria). These results suggest that brown algae acquired *PMM* from eukaryotic hosts (Fig. 2).

Several genes belonged to the *GMD/UGD* superfamily in brown algae, and these clustered into two separate branches. Brown algal GMD formed the “GMD branch” with archaea, bacteria, and cyanobacteria GMD, while the “UGD branch” included archaea, bacteria, cyanobacteria, and eukaryotic UGD (Fig. 3). A phylogenetic analysis showed that *GMD* of brown algae was likely inherited from bacteria via HGT. Furthermore, *GMD* in the ancestor of brown algae underwent gene duplication, resulting in two copies (e.g. *GMD1* and *GMD2* in *Saccharina*), followed by subsequent duplication events within some species (e.g. *GMD2* and *GMD3* in *E. siliculosus*). The brown algal *UGD* had a different origin from their secondary endosymbiotic hosts, as evidenced by their close relationships to oomycete homologs. The different origins and evolutionary patterns of *GMD* and *UGD* suggest different functions and regulatory mechanisms (see GMD/UGD function and transcriptional regulation below).

Among eukaryotic algae, only brown algae contained *MC5Es*, which are homologs of *AlgY* and *AlgE1*-*7* of *Azotobacter vinelandii* and bacterial *AlgG.* All MC5Es and AlgGs (including those of *Azotobacter*) formed a lineage (PP = 1.0), indicating that *MC5E* is more closely related to *AlgG* than *AlgE*, and brown algal *MC5E* originated from bacteria via HGT (Fig. 4). Brown algae contained a large family of *MC5Es*, and these could be divided into five large groups (Group I–V), with more than one type in each species (Figure S4). Duplication of the gene group occurred in the common ancestor of brown algae and independent duplication events later occurred within species (e.g. *MC5E1* and *MC5E2* from *Saccharina sculpera* of Group I; Figure S4). In particular, only *E. siliculosus* had the special gene type of Group V, and other brown algae, such as *Saccharina,* contained only four types (Group I–IV).

In the consensus tree of *GM46D*, brown algae and diatoms clustered independently and were the sister taxon of oomycetes (Figure S1). *GM46D* was absent in rhodophytes; therefore, secondary endosymbiotic brown algal *GM46D* originated from secondary endosymbiotic hosts. Similar to *GM46D*, *GFS* also lacked the rhodophytal homolog, indicating that brown algal *GFS* was also a non-rhodobiont-derived gene (Figure S2). Phaeophyceae algae and haptophytes formed a well-supported clade with oomycetes, fungi, and protozoa, indicating their relationship with secondary endosymbiotic hosts.

### Characterization and confirmation of the functions of alginate and fucoidan biosynthesis genes

In this study, a subset of alginate and fucoidan biosynthesis genes detected in the *Saccharina* transcriptome was chosen to verify their encoding enzyme activity. One *Saccharina* MPI (SjaMPI4) exhibited the same function as MPG, SjaPMM had both PMM and PGM activity, and UGD (SjaUGD and EsiUGD) activities were similar to GMD activity. This was the first functional analysis of these enzymes which involved in the alginate and fucoidan biosynthesis pathways in brown algae.

**Fig. 5:**
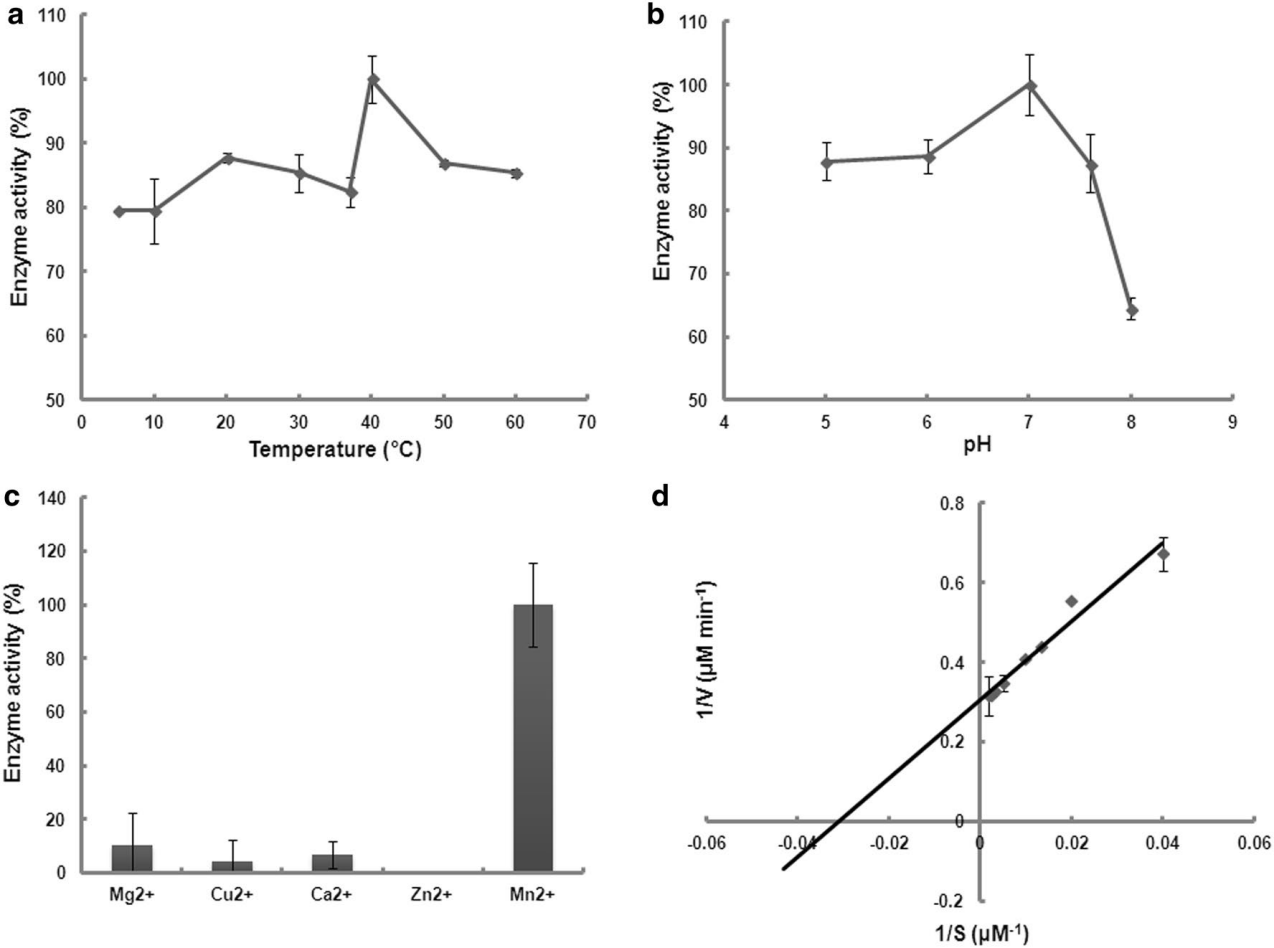
Mannose-1-phosphate guanylyltransferase (MPG) activity of SjaMPI4. Effects of temperature (**a**), pH (**b**), and the presence of metal ions (**c**) on activity. **a** Enzyme activity at 40 °C was set to 100%. **b** Enzyme activity at pH 7.0 was set to 100%. **c** Enzyme activity in the presence of Mn^2+^ was set to 100%. **d** The double reciprocal plot of enzyme activity for GDP-mannose. Data represent mean ± SD of four independent experiments

It is possible that the function of *MPG*, which was not detected in the brown algae genome, was adopted by algal *MPI*. Three full-length *MPI* genes from *Saccharina* were isolated, and enzyme assay confirmed that *SjaMPI4* encodes functional MPG protein. The optimal temperature and pH value for MPG activity were 40 °C and 7.0 (Fig. 5a, b), respectively. A buffer with high alkalinity significantly inhibited the enzyme activity. Divalent ions, such as Mg^2+^, Mn^2+^, Ca^2+^, and Cu^2+^, promoted enzymatic activity, particularly Mn^2+^ (Fig. 5c). A specific activity of 54.68 nanokatal and a *K*_m_ of 32.44 μM were observed using Mg^2+^ at a final concentration of 2 mM (Fig. 5d).

**Fig. 6:**
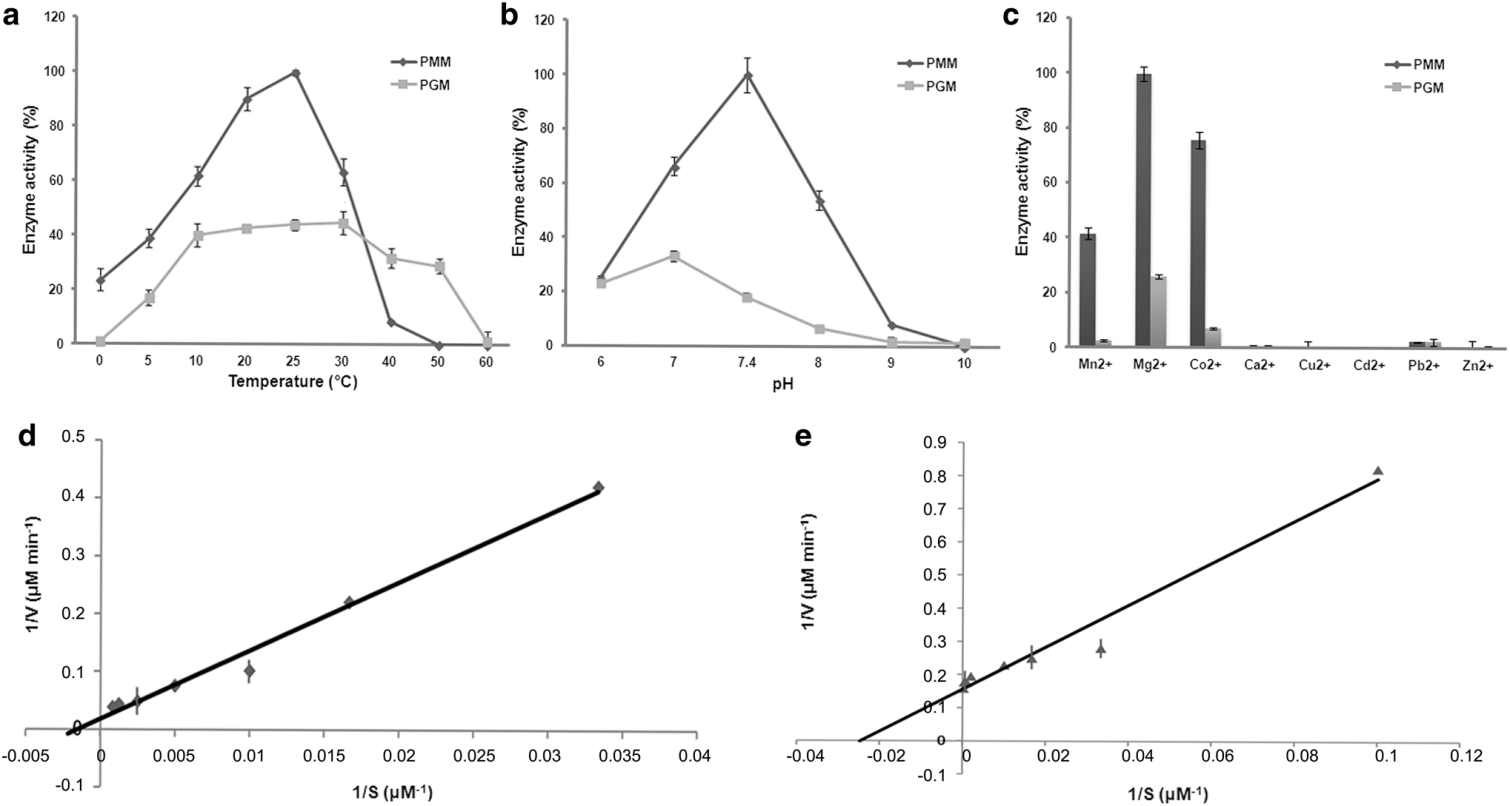
Phosphomannomutase and phosphoglucomutase activity of SjaPMM. Effects of temperature (**a**), pH (**b**), and the presence of metal ions (**c**) on activity. **a** PMM activity at 25 °C was set to 100%. **b** PMM activity at pH 7.4 was set to 100%. **c** PMM activity in the presence of Mg^2+^ was set to 100%. **d** The double reciprocal plot of enzyme activity for mannose-1-P. **e** The double reciprocal plot of enzyme activity for glucose-1-P. Data represent mean ± SD of four independent experiments

Based on enzyme activity analysis, SjaPMM was a bifunctional enzyme. The optimal temperature for phosphomannomutase and phosphoglucomutase enzyme activity were 25 and 30 °C, and the optimal pH were 7.4 and 7.0, respectively (Fig. 6a, b). Mg^2+^ and Co^2+^ significantly promoted the enzymatic activity of both PMM and PGM (Fig. 6c). The specific activity of PMM (980.53 nanokatal) was significantly higher (almost 10 times) than that of PGM (107.52 nanokatal). However, the binding capacity of glucose-1-phosphate (*K*_m_ = 41.04 μM) was almost 17 times higher than that of mannose-1-phosphate (*K*_m_ = 699.41 μM) (Fig. 6d, e).

Three *S. japonica GMD/UGDs* (*SjaGMD1*, *SjaGMD2*, and *SjaUGD*) and three *E. siliculosus GMD/UGDs* (*EsiGMD2*, *EsiGMD3*, and *EsiUGD*; except *EsiGMD1*, which had been studied previously) were expressed and functionally verified, and all enzymes had GMD activity with different specific activities and optimal reaction conditions (Table 1). The optimal temperature of EsiGMDs was maintained at 30–37 °C, while that of the two SjaGMDs changed (the optima for SjaGMD1 was 20 °C and for SjaGMD2 was 37–40 °C), indicating that the temperature adaptabilities of different SjaGMD homologs were wider than that of EsiGMDs. The optimal reaction conditions of EsiGMD2 and EsiGMD3 were similar, but the specific activity of EsiGMD2 was almost 10 times that of EsiGMD3. Similarly, the specific activity of SjaGMD2 was much higher than that of SjaGMD1. In addition, SjaUGD and EsiUGD both had GMD function. SjaUGD, with a wider optimal temperature range (25–50 °C), had a specific activity about 5 times that of EsiUGD.

**Table 1:** Biochemical characterization of UDP-glucose/GDP-mannose dehydrogenases in *Saccharina* and *Ectocarpus*

Enzymes	Specific activity (nanokatals mg^−1^)	Temp (°C)	pH	GDP-M		NAD	
				*k*_cat_ (s^−1^)	*k*_m_ (mM)	*k*_cat_ (s^−1^)	*k*_m_ (mM)
SjaGMD1	0.19	20 (>30 activity lost)	8.75	0.19	812.3	0.038	112.5
SjaGMD2	6.80	37–40 (>50 activity lost)	8.75–9	2.99	107.836	2.70	197.25
SjaUGD	6.37	25–50 (>50 activity lost)	8–9	27.59	3283.0	5.6	380.2
EsiGMD2	6.6	30–37 (>50 activity lost)	8.75–9	2.22	971.6	1.28	79.6
EsiGMD3	0.49	30–37 (>50 activity lost)	8.75	0.11	1433.97	0.18	43.49
EsiUGD	1.21	40–50	9	0.72	36.86	0.60	41.99
EsiGMD1*	3.3	30 (>30 activity lost)	8.75–9	0.21	95	0.21	86

* Tenhaken et al. (2011)

In terms of enzyme catalytic reaction efficiency and substrate binding capacity, there were large differences among GMD/UGD family members (Table 1). First, the catalytic efficiency of SjaUGD was highest, and was approximately 4.5–250 times that of *Ectocarpus* GMD/UGD enzymes. Secondly, the preferences of various genes for the two substrates (GDP-mannose and NAD) varied. The binding capacities of 3 SjaGMD1 and SjaUGD on GDP-mannose were lower than those on NAD, and the opposite trend was observed for SjaGMD2. In *Ectocarpus*, the binding capacities of three EsiGMDs on GDP-mannose were lower than those on NAD, and EsiUGD exhibited the opposite pattern. Finally, SjaGMD1 and EsiGMD3, with the lowest specific activities among all *Saccharina* and *Ectocarpus* UGD/GMD enzymes (Table 1), also had the lowest catalytic efficiencies.

**Fig. 7:**
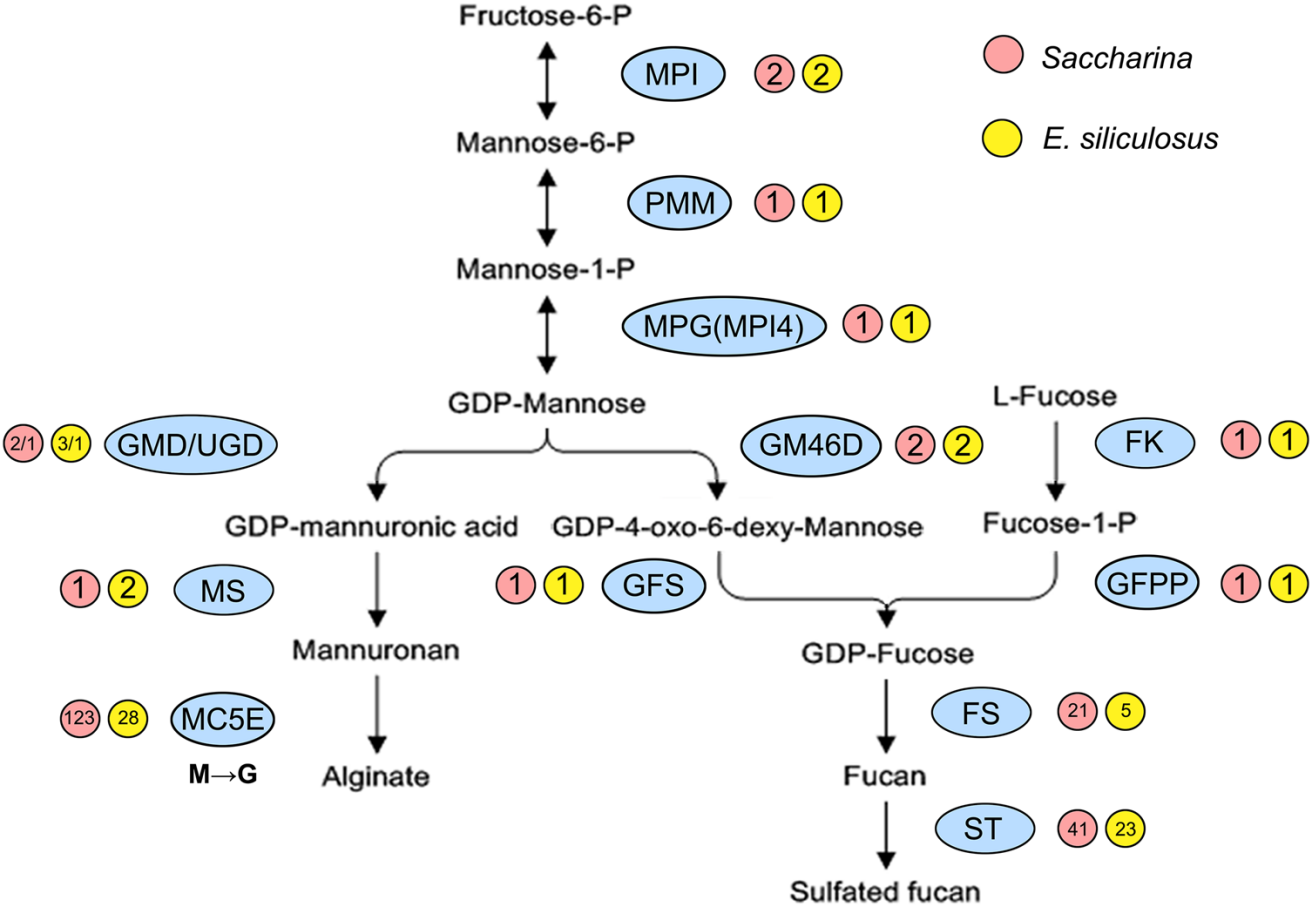
Biosynthetic routes of alginate and fucoidan in algae. *MPI4* was the actual *MPG* gene. The *GMD/UGD* gene family members all encoded enzymes with GMD activity. MC5E catalyzes the isomerization from mannuronic acid (M) to guluronic acid (G) at the alginate polymer level. *MPI* mannose-6-phosphate isomerase, *PMM* phosphomannomutase, *MPG* mannose-1-phosphate guanylyltransferase, *GMD/UGD* GDP-mannose/UDP-glucose 6-dehydrogenase, *MS* mannuronan synthase, *MC5E* mannuronate C5-epimerase, *GM46D* GDP-mannose 4,6-dehydratase, *GFS* GDP-fucose synthetase, *FK* fucokinase, *GFPP* GDP-fucose pyrophosphorylase, *FS* fucosyltransferase, *ST* sulfotransferase

The biosynthetic routes of alginate and fucoidan in brown algae were reconstructed according to the above functional results. Two pathways shared 3 upstream genes (*MPI*, *PMM*, and *MPG* which was actually *MPI4*) that catalyzed the conversion of fructose-6-P to GDP-mannose, with brown algal *MPI4* acquiring the function of *MPG.* Then, GDP-mannose was used to synthesize alginate and fucoidan separately, and enzymes encoded by *GMD* and *UGD* performed the functions of GMD (Fig. 7). By scanning the genome data, *Saccharina* and *Ectocarpus* contained almost the same number of copies of the upstream monomer substrates (GDP-mannuronic acid and GDP-fucose) synthesis genes (e.g. *MPI*, *PMM*, and *GM46D*); however, the downstream glycosyltransferase and modification genes were much more expanded in *Saccharina* than *Ectocarpus* (e.g. *MC5E*, *FS,* and *ST*).

### Expression differences in alginate and fucoidan biosynthesis

The transcriptional regulation of alginate and fucoidan genes is very complex in *Saccharina*. First, various abiotic factors altered gene expression levels and promoted adaptation to the constantly changing environment. Second, the genes/families involved in the synthesis pathways all contained constitutively expressed member(s), while the downstream gene family members were differentially transcribed at various stages of development and in different tissue structures. Finally, the overall expression profiles of genes involved in the two routes differed between the gametophyte and sporophyte generations.

**Fig. 8:**
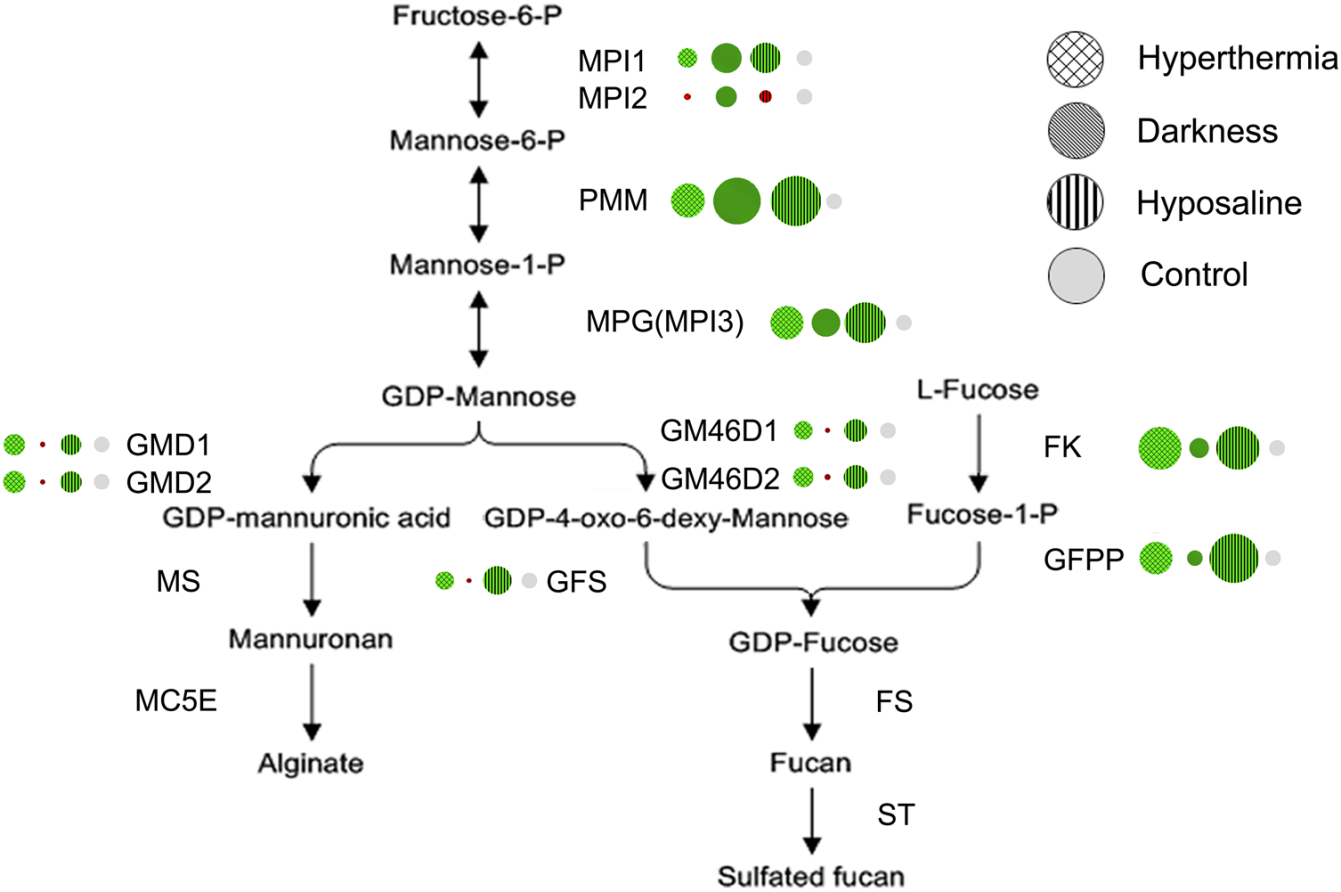
Summary of gene expression under abiotic stresses (hyperthermia, hyposaline, and continuous darkness). Genes in *green* exhibited increased expression and genes in *red* exhibited decreased expression. The *circular area* represents the relative expression level

Droplet digital PCR experiments were conducted using algal samples under short-term abiotic stresses and 72 h of darkness. A similar trend was observed under both hyperthermia and hyposaline treatments, all upstream monomer synthesis gene transcripts (except *SjaMPI2*) of the two routes (including the salvage pathway of fucoidan) increased (Fig. 8). For instance, *SjaPMM* showed a 4.93-fold induction in the hyperthermia treatment and 10.0-fold induction in the hyposaline treatment (Table S2). Different results were obtained in continuous dark conditions. The expression levels of 3 shared genes increased (Fig. 8, Table S2). However, after the common substrate GDP-mannose flowed in two directions, the expression levels of the underlying genes (*SjaGMD1*, *SjaGMD2*, *SjaGM46D1*, *SjaGM46D2,* and *SjaGFS*) all decreased dramatically to 0.10–0.14 times that of the initial levels. Interestingly, gene expression in the fucoidan alternative salvage pathway increased slightly.

Gene expression levels were then examined at various developmental stages and in different tissues of *Saccharina* (3 gametophytes and 9 sporophytes samples) based on transcriptome data. The upstream monomer synthetic genes were all constitutively expressed, except *SjaMPI2* (Table S3). Downstream glycosyltransferase and modification genes with a large number of homologs (only genes with full-length transcripts were studied) exhibited different expression patterns (Table S3). For the *SjaMC5E* gene family, some members were expressed constitutively (e.g. *SjaMC5E3*, *SjaMC5E6,* and *SjaMC5E10*), while some were expressed specifically (e.g. *SjaMC5E12* was expressed only in gametophytes and *SjaMC5E5* was expressed only in sporophytes) (Figure S5). Similar patterns were observed in *SjaFS* and *SjaST* superfamilies (Table S3).

The average gene expression levels in the gametophyte and sporophyte generations were further compared. The shared pathway genes were expressed at slightly higher levels in sporophytes than in gametophytes, but the vast majority of alginate synthesis-specific genes (12/16 genes) were expressed at much higher levels in sporophytes. For instance, *SjaGMD2* transcription displayed an 11.8-fold increase; *SjaMC5E1* increased by 6.3 times, and *SjaMC5E4*, *SjaMC5E5*, and *SjaMC5E8* were only expressed in sporophyte stages (Figure S5, Table S3). However, the expression of fucoidan biosynthesis genes did not show a consistent trend (only 24 genes of 45 were expressed at higher levels in sporophytes) (Table S3).

## Discussion

### Establishment and functioning of complete alginate and fucoidan pathways conferred uniqueness to brown algae compared with other eukaryotic algae

#### Integration of genes from separate sources contributed to pathway evolution in brown algae

**Fig. 9:**
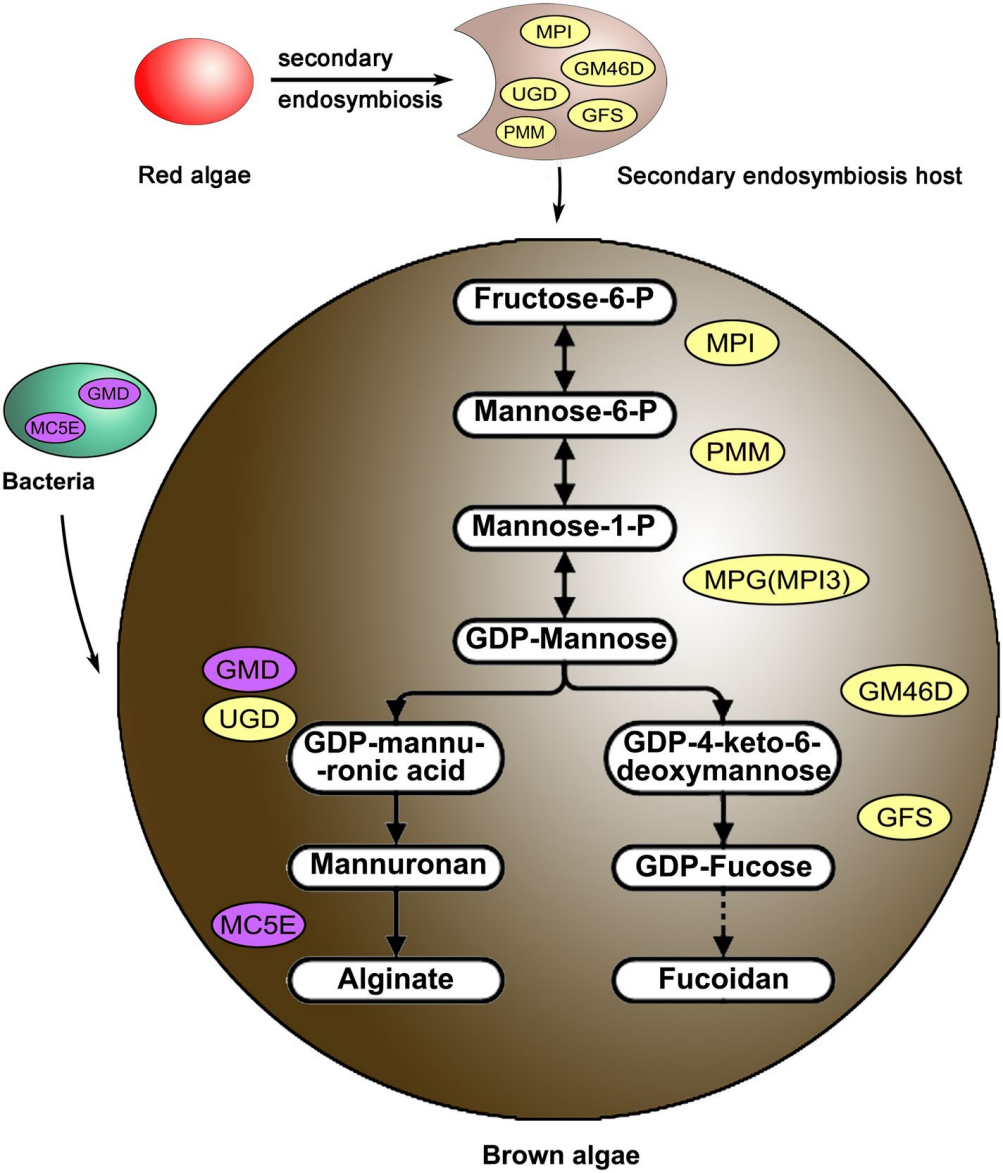
Origins of alginate and fucoidan synthesis genes in algae. The genes in *yellow* were derived from secondary endosymbiotic eukaryotic hosts and genes in *purple* were derived from bacteria via horizontal gene transfer

Alginate and fucoidan synthetic pathway genes in brown algae had different origins. Some genes (e.g. *GMD* and *MC5E*) were acquired by HGT from bacteria, similar to previous results for *E. siliculosus* and *S. japonica* (Michel et al. 2010; Ye et al. 2015). Some genes (e.g. *MPI*, *GM46D,* and *GFS*) originated from secondary endosymbiotic eukaryotic hosts, like the eukaryotic ancestral origin of *E. siliculosus* genes (Michel et al. 2010). However, *PMM* was inherited from endosymbiotic hosts, different from the cyanobacterial origin of this gene in *E. siliculosus* (Michel et al. 2010). This difference may be explained by the selection of the source sequences used in the phylogenetic analysis, and a more extensive analysis of many species may provide a more accurate assessment of the origin and evolution of these pathway genes. A similar situation occurred for brown algal mannitol-1-phosphatase gene (involved in mannitol synthesis pathway) analysis. This gene was first suggested to be imported by HGT from actinobacteria (Michel et al. 2010), then was shown to originate from the nonphotosynthetic eukaryotic hosts using more comprehensive assessments across various algal lineages (Tonon et al. 2017). In addition, *UGD* was added to the alginate biosynthesis pathway via endosymbiotic hosts. Therefore, the brown algae alginate and fucoidan pathways exhibited a mosaic pattern of gene origins (Fig. 9), similar to algal C_4_-related genes (Chi et al. 2014).

#### Evolution of gene function resulted in complete and effective pathways in brown algae

The enzyme activities of brown algal MPI, PMM and GMD (and UGD) were confirmed (Fig. 6; Table 1), verifying the authenticity of the pathways determined by bioinformatics approaches. Previously, no *MPG* gene was detected in algae by genomic and transcriptomic analyses. In this study, the protein encoded by *MPI4* was confirmed to have MPG activity (Fig. 5), similar to the bacterial AlgA (MPI) with MPG function (Jensen and Reeves 1998; Roux et al. 2007; Griffin et al. 1997; Sousa et al. 2007), and this enzyme was integrated into the synthetic alginate and fucoidan pathways in brown algae. A phylogenetic analysis showed that the duplication of *MPI* occurred in a coancestry period after phaeophytes diverged from diatoms (Fig. 5), and *MPI4* diverged from *MPI1* and *MPI2*. *MPI4* functional evolution after gene duplication was a key event in alginate and fucoidan synthesis pathway evolution, similar to results obtained for the biosynthesis pathway of plant starch in which the molecular evolution of a multi-copy gene was accompanied by functional differentiation (Nougué et al. 2014). In addition, only one *MPI* gene has been observed in diatoms *T. pseudonana* and *P. tricornutum*, red algae *Cyanidioschyzon merolae* and *Galdieria sulphuraria*, and in some green algae like *Ostreococcus lucimarinus*, *Micromonas* sp., and *Chlorella variabilis*; these species all lack an *MPG* homolog (Armbrust et al. 2004; Bowler et al. 2008; Matsuzaki et al. 2004; Schönknecht et al. 2013; Palenik et al. 2007; Blanc et al. 2010). Accordingly, these pathways may be specific to brown algae after they diverged from other heterokonts.

#### All pathway genes had constitutively expressed member(s) for the synthesis of cell wall polysaccharides to meet fundamental growth and development needs of brown algae

During the development of *Saccharina*, zygotes divide continuously from a single cell to form thallus sporophytes, which exhibit consistent increases in length, width, and thickness. Therefore, alginate and fucoidan, the major cell wall contents, are needed constantly. The pathway genes, whether single- or multi-copy, all contain constitutively expressed members at various developmental stages and in different tissues, and under various environmental conditions. Therefore, brown algae consistently synthesize alginate and fucoidan at basal levels. However, *SjaMPI2* showed a different transcriptional pattern under abiotic stress and in various stages of development and tissues. Interestingly, we could not detect any MPI or MPG activities of the SjaMPI2 protein. We inferred that during brown algae differentiation, *MPI* undergoes duplication. One of the duplicates (*SjaMPI1*) may retain its original function, another member (*SjaMPI4*) evolved MPG function, and the third copy (*SjaMPI2*) may accumulate molecular changes and take on a totally new function that was not present in the ancestral gene, which is also called neofunctionalization (Rastogi and Liberles 2005). This phenomenon also had been found in the UDP-glucose pyrophosphorylase (*UGP*)*/PGM* genes in brown algae (Chi et al. 2015), and further research is needed to understand the true function of *SjaMPI2*.

### The complex regulation of alginate and fucoidan biosynthesis was a driving force for the sophisticated system evolution in brown algae

#### Gene transcriptional regulation differences and downstream gene expansion were conducive to brown algae adaptation to the surrounding environment and the large structure evolution

Gene expression under diverse abiotic stresses showed significant differences. All upstream monomer synthesis gene transcripts (except *SjaMPI2*) increased under both hyperthermia and hyposaline (Fig. 8). In these situations, more polysaccharides may be synthesized to induce rapid morphological changes in brown algae as a strategy to increase resistance to these stresses, similar to the effects of temperature or salinity changes on brown algae morphogenesis (Lobban and Harrison 1994; Pereira et al. 2011). However, under continuous dark conditions, the expression levels of 3 shared upstream genes increased while the underlying genes (*SjaGMD1*, *SjaGMD2*, *SjaGM46D1*, *SjaGM46D2,* and *SjaGFS*) all decreased dramatically (Fig. 8). As the shared genes are all functionally reversible, brown algae may reversely synthesize fructose-6-*P* and further generate other basic metabolites such as mannitol, which has an inverse relationship to alginate and fucoidan with respect to accumulation (Kaliaperumal and Kalimuthu 1976; Ji 1963). Gene copies of downstream genes showed an obvious advantage in *Saccharina* compared to *Ectocarpus* (Fig. 7). In addition, there were a large number of downstream genes that are specifically expressed only in particular stages (Figure S5, Table S3). Because diverse *MC5Es* may modify specific MG blocks (Svanem et al. 2001), *MC5E* expression differences may explain alginate diversities with various M/G ratios among algal developmental stages and tissues. Different M/G ratios changed the toughness of the *Saccharina* thallus, enabling algae to adopt a flexible body that is particularly suited for constant water fluxes resulting from tides, waves, and local currents. The synthesis of polysaccharides with different structures and characteristics is necessary for the flexibility, strength, and antibacterial properties of brown algae (Koehl and Wainwright 1977). These properties could have promoted brown algae evolution. In addition, gene numbers in *Saccharina* (“Rongfu”) were not the same as those observed in the published *S. japonica* genome, including gene numbers for *MC5Es* (123 in “Rongfu” versus 105 in *S. japonica*) and *STs* (41 in “Rongfu” versus 24 in *S. japonica*). These differences may be explained by the different sequencing samples or incomplete sequencing. The sequencing sample “Rongfu” is a high-yielding cultivated variety with the parental background of *S. japonica* and *S. latissima* (Zhang et al. 2011).

#### Higher expression of alginate genes (not fucoidan genes) and increased polysaccharide accumulation in sporophytes increased thallus strength and toughness

*Saccharina* have heteromorphic haploid–diploid life cycles with a macroscopic thallus sporophyte and microscopic gametophyte generation, and the filamentous gametophyte is similar to some brown algae, with an isomorphic haploid–diploid filamentous generation, such as that of its close relative *Ectocarpus* (Cock et al. 2014; Bartsch et al. 2008). Therefore, the comparison between *Saccharina* sporophytes and gametophytes may provide an explanation for the evolution of complex systems in brown algae. The significantly higher expression of alginate synthesis genes in sporophytes than gametophytes suggests that sporophytes synthesize alginate at higher quantities and form more sophisticated structures compared with those of gametophytes. This is consistent with our alginate content analysis in *Saccharina*, in which the dry weight of alginate contents was much higher in sporophytes of 13.8% (±0.3) than in female gametophytes of 2.2% (±0.2) (*P* < 0.01). Meanwhile, fucoidan biosynthesis gene expression in gametophytes and sporophytes only showed a slight difference, consistent with the content analysis showing 4.7% (±0.1) fucoidan in female gametophytes and 6.7% (±0.2) in sporophytes. Furthermore, the overall specific activities of *Saccharina* GMD/UGD were higher than those of *Ectocarpus* homologs. In brown algae, alginate seems to play a more important role in cell structure support than fucoidan, with the former acting as the skeletal fibers and forming the alginate network in brown algae cell wall, and the latter is more like a filling component (Gurvan et al. 2010; Bartsch et al. 2008). Therefore, the high expression levels of alginate (not fucoidan) synthesis genes can meet the constant growing demands of the thallus, and may play potential roles in complex multicellularity evolution of brown algae.

In conclusion, in this study, we conducted a comprehensive bioinformatic analysis, together with phylogenetic analysis, demonstrating that genes from separate sources were integrated in alginate and fucoidan pathway evolution in brown algae. Enzyme assays suggested that the predicted corresponding genes had relevant functions, and *MPI4* evolved to possess *MPG* function. Gene expression analysis showed that all pathway genes/families had constitutively expressed member(s) to maintain fundamental synthesis of these cell wall polysaccharides. Further analysis found an obvious advantage of downstream gene copies in *Saccharina* compared to *Ectocarpus*, and transcriptional expression differences in various *Saccharina*. These results suggested the gene function differentiation, enzyme characterization, and gene expression regulation differences conferred uniqueness to brown algae compared with other eukaryotic algae, and have an important ecophysiological significance for environment adaptation and complex multicellularity evolution. In industry, alginate and fucoidan are mainly produced by extraction from brown algae (Cunha and Grenha 2016; Li et al. 2017). However, some extraction methods may alter the natural structure, affecting the bioactivity and physicochemical properties (Qiu et al. 2006). Clarifying the evolution and function of these synthesis pathway genes can provide theoretical and experimental bases for the artificial synthesis of these bioactive compounds in vitro, and may also provide a basis for breeding research in the future.

## Electronic supplementary material

Below is the link to the electronic supplementary material.

Supplementary material 1 (DOCX 921 kb)

Supplementary material 2 (XLS 164 kb)
