# Supplementary material for: Functional genomics analysis reveals the biosynthesis pathways of important cellular components (alginate and fucoidan) of Saccharina
Source: Curr Genet. 2017 Aug 19;64(1):259–73. doi: 10.1007/s00294-017-0733-4 (PMC5778160; doi:10.1007/s00294-017-0733-4)
Supplement: Supplementary file 1 — Supplementary material 1 (DOCX 921 kb) [file 294_2017_733_MOESM1_ESM.docx]

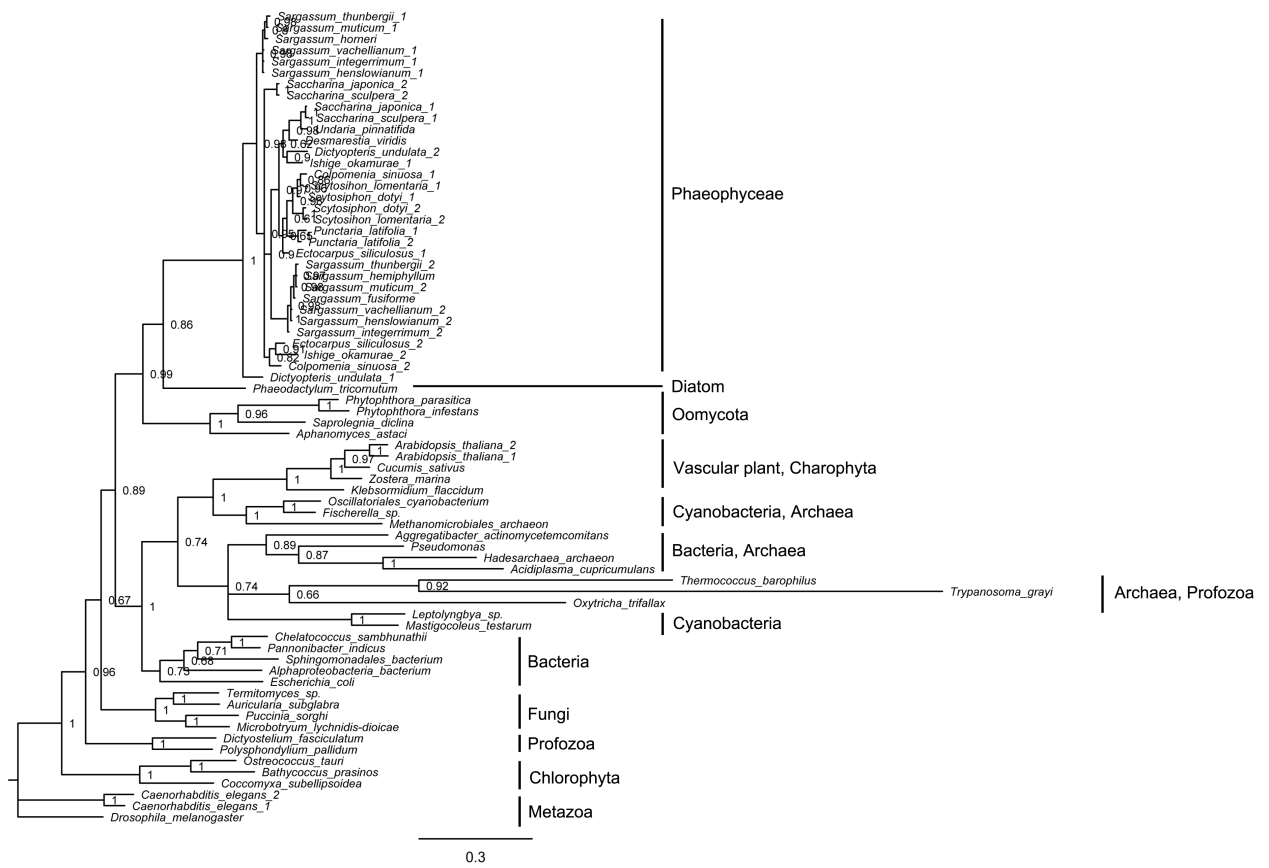


Figure S1. Bayesian phylogenetic tree based on the translated amino acids of GM46D from various algal, plant, oomycete, fungal, bacterial, cyanobacterial, and archaeal taxa, with bootstrap values (when >50%) indicated at the nodes. All GM46D sequences were obtained from the GenBank or OneKP databases (Table S1).


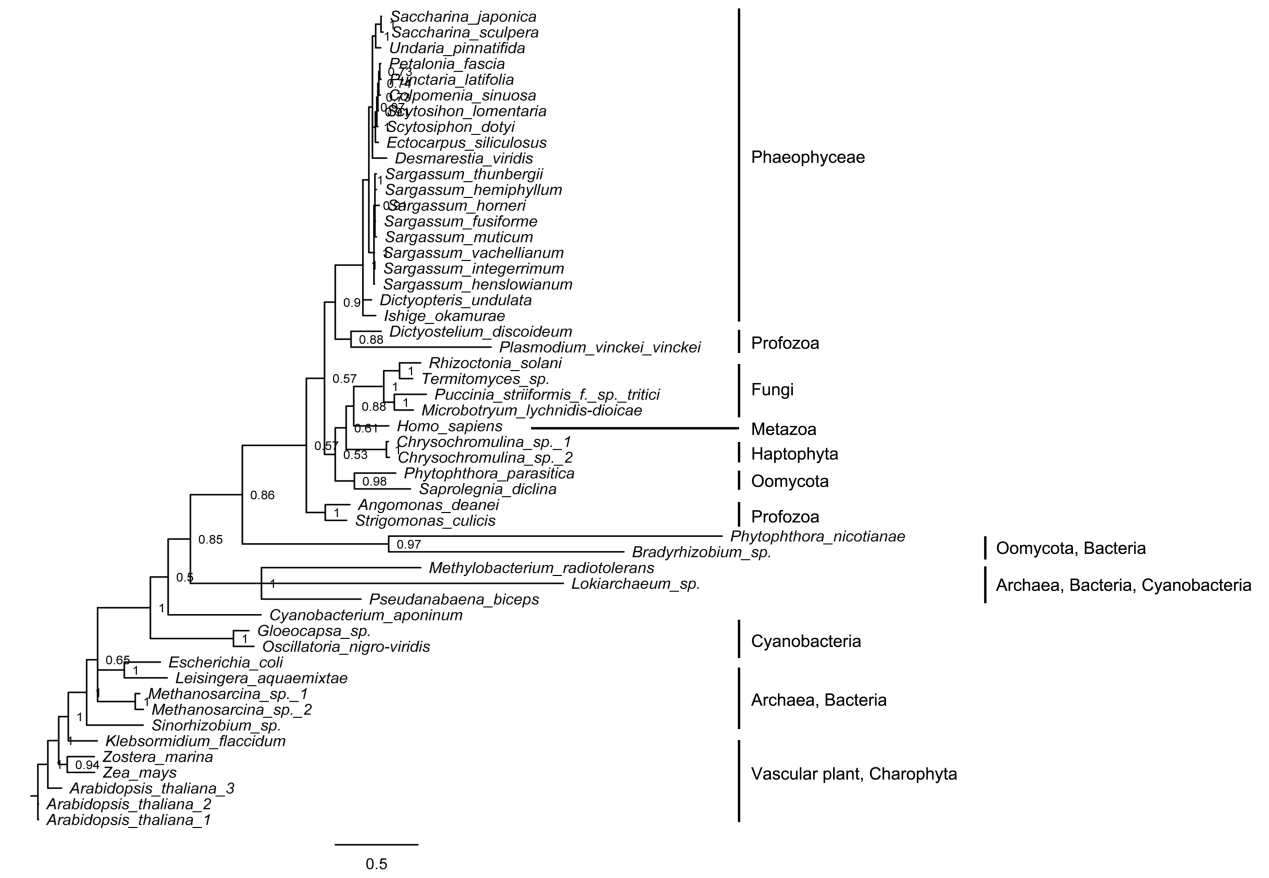


Figure S2. Bayesian phylogenetic tree based on the translated amino acids of GFS from various algal, plant, oomycete, fungal, bacterial, cyanobacterial, and archaeal taxa, with bootstrap values (when >50%) indicated at the nodes. All GFS sequences were obtained from the GenBank or OneKP databases (Table S1).


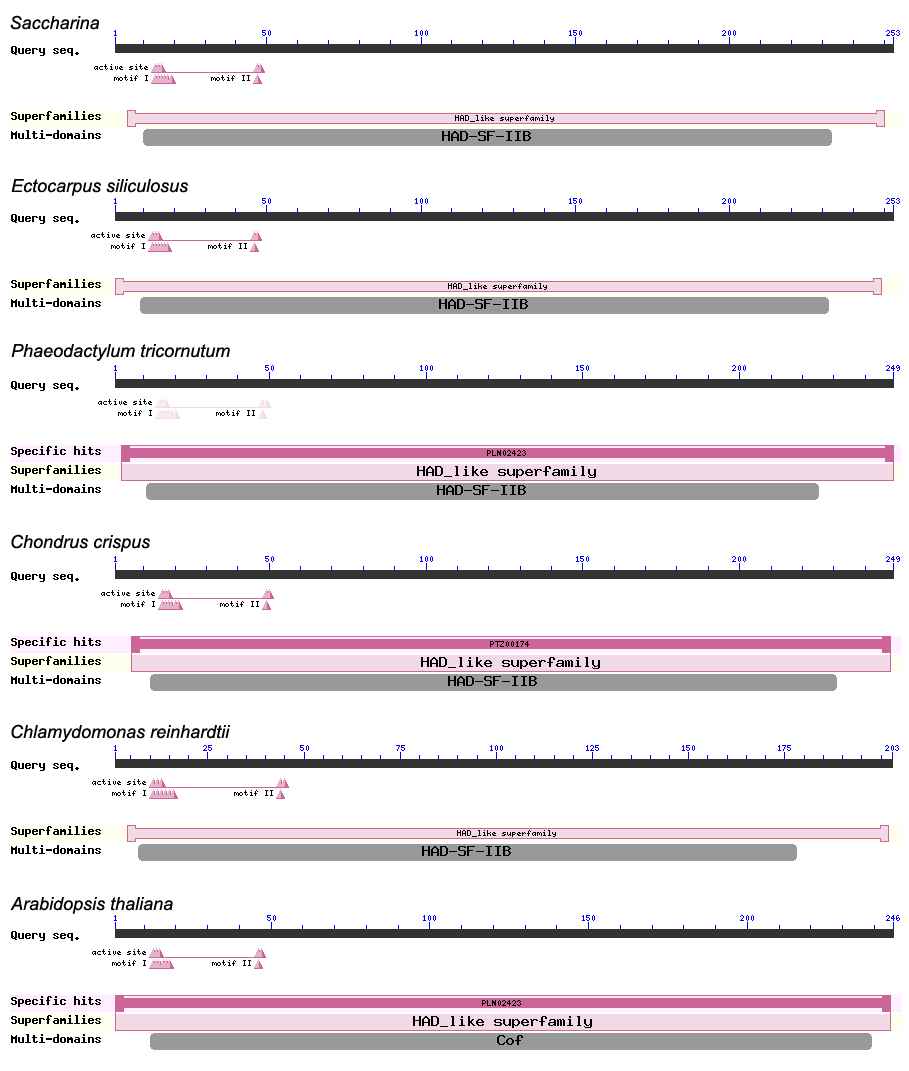


Figure S3. Conserved domain analysis of eukaryotic PMM using the online “Conserved Domain Search Service” (<https://www.ncbi.nlm.nih.gov/Structure/cdd/wrpsb.cgi>). The haloacid dehalogenase (HAD) superfamily includes carbon and phosphorus hydrolases such as phosphomannomutase.


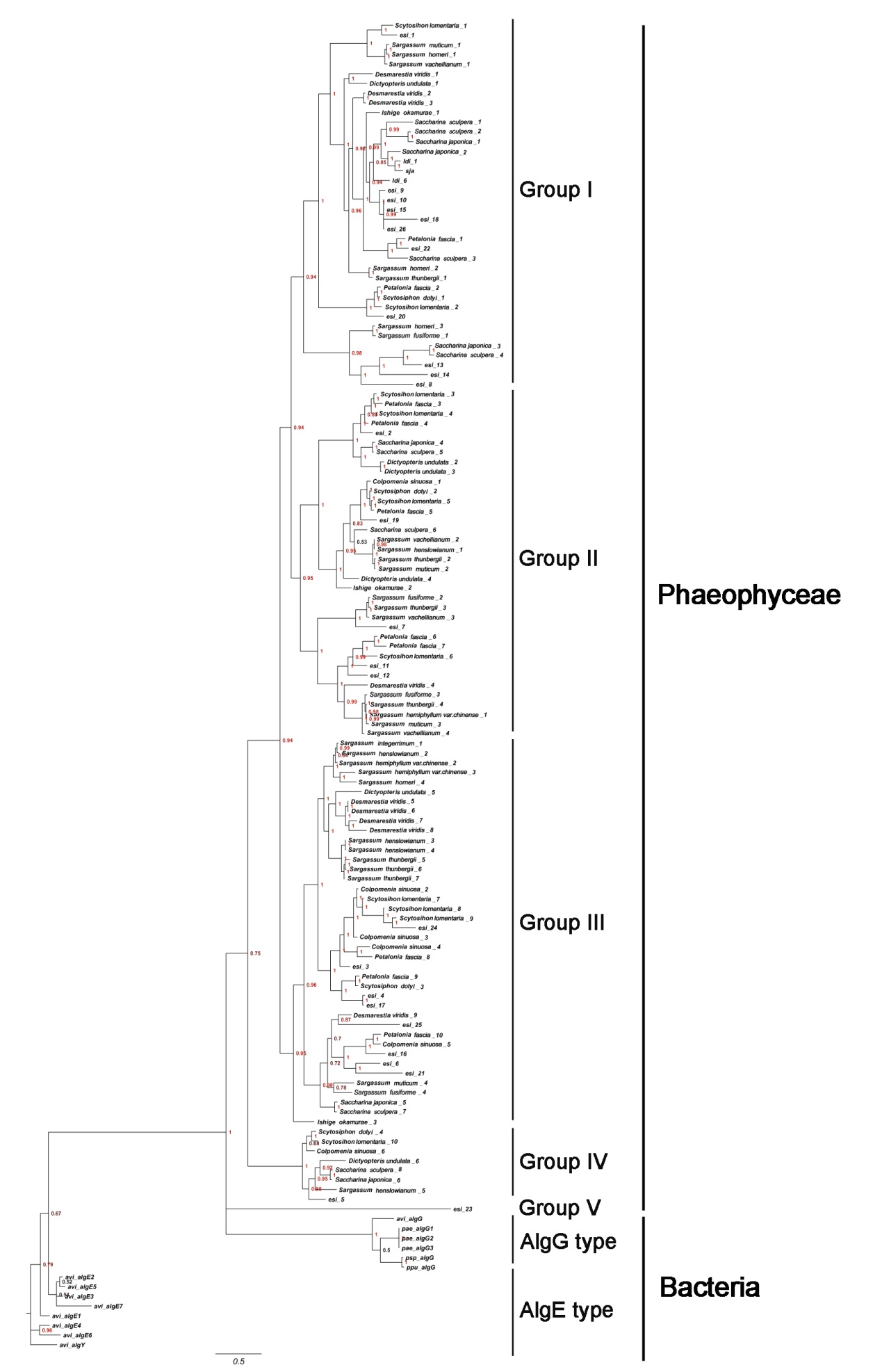


Figure S4. The detailed phylogenetic tree of MC5E.


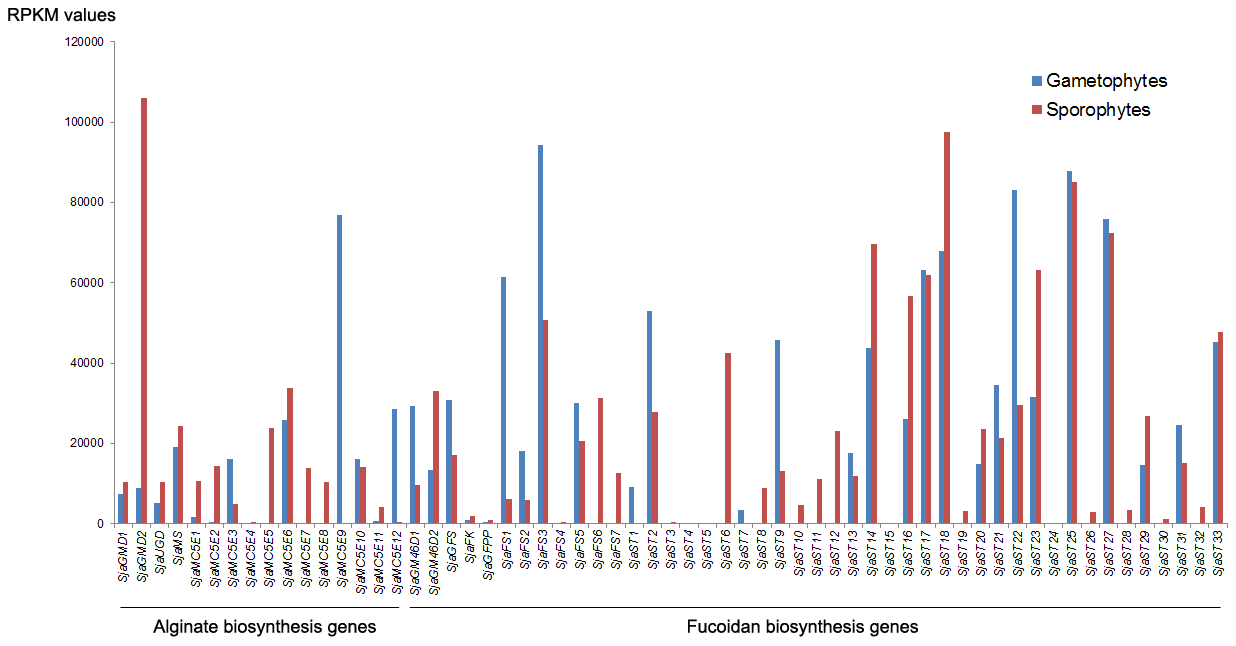


Figure S5. Expression differences of specifically expressed gene family members in alginate and fucoidan biosynthesis pathways between gametophyte and sporophyte stages. 75% (12/16 genes) of alginate synthesis-specific genes were expressed at much higher levels in sporophytes, but only 53.3% (24/45 genes) of fucoidan synthesis-specific genes showed consistent trend.
